# Ultrafast spontaneous emission of copper-doped silicon enhanced by an optical nanocavity

**DOI:** 10.1038/srep05040

**Published:** 2014-05-23

**Authors:** HISASHI SUMIKURA, EIICHI KURAMOCHI, HIDEAKI TANIYAMA, MASAYA NOTOMI

**Affiliations:** 1 NTT Basic Research Laboratories, NTT Corporation, 3-1 Morinosato Wakamiya, Atsugi, Kanagawa 243-0198, Japan; 2 NTT Nanophotonics Center, NTT Corporation, 3-1 Morinosato Wakamiya, Atsugi, Kanagawa 243-0198, Japan

## Abstract

Dopants in silicon (Si) have attracted attention in the fields of photonics and quantum optics. However, the optical characteristics are limited by the small spontaneous emission rate of dopants in Si. This study demonstrates a large increase in the spontaneous emission rate of copper isoelectronic centres (Cu-IECs) doped into Si photonic crystal nanocavities. In a cavity with a quality factor (*Q*) of ~16,000, the photoluminescence (PL) lifetime of the Cu-IECs is 1.1 ns, which is 30 times shorter than the lifetime of a sample without a cavity. The PL decay rate is increased in proportion to *Q*/*V*_c_ (*V*_c_ is the cavity mode volume), which indicates the Purcell effect. This is the first demonstration of a cavity-enhanced ultrafast spontaneous emission from dopants in Si, and it may lead to the development of fast and efficient Si light emitters and Si quantum optical devices based on dopants with efficient optical access.

In photonic and quantum optical studies, dopants in silicon (Si) have attracted attention as light emitters and quantum bits (qubits). This is because Si is a promising low-loss optical platform for photonic integrated circuits^1^,^2^,^3^,^4^,^5^,^6^. In quantum optics, dopants in Si have been well studied with a view to realizing a long-lived qubit because a high-quality Si crystal provides a pure quantum system of dopants with less dephasing^7^,^8^,^9^,^10^,^11^. In addition, these dopant-based qubits will be implemented in Si quantum optical circuits consisting of low-loss optical waveguides and entangled photon sources^12^,^13^. However, the potential use of dopants in Si devices has been limited by their poor emission characteristics and inefficient optical access to dopants, which result from the weak interaction between light and dopants. Since the weak interaction is observed as the small spontaneous emission rate of dopant-related radiative centres in Si, the enhancement of their spontaneous emission rate is necessary to improve light-dopant interaction and optical characteristics of dopant-based Si devices.

The spontaneous emission rate of dopants in Si is increased by the Purcell effect in optical cavities^14^. If the emission linewidth of the dopants is smaller than the cavity linewidth, the Purcell enhancement of the emission rate is proportional to *Q*/*V*_c_, where *Q* and *V*_c_ are the quality factor and the mode volume of the optical cavity, respectively^15^. This means that the high *Q* and small *V*_c_ of nanocavities make it possible to obtain a large increase in the emission rate of radiative centres doped in Si. The Purcell effect is especially pronounced in Si photonic crystal (PhC) nanocavities because ultrafine Si processes enable us to fabricate Si PhC nanocavities with a high *Q* of over 10^6^ and an ultrasmall *V*_c_ of less than 0.1 μm^3^ 16,17,18. In addition, Si PhC nanocavities are suitable for integration in Si photonic circuits and efficient optical access through optical waveguides.

The Purcell effect of the emission from Si itself or radiative defects in Si have already been reported^19^,^20^,^21^. Most of the studies have reported the enhancement of photoluminescence (PL) intensity by optical cavities. However, intensity enhancement is insufficient to prove the Purcell effect because the PL intensity is not only increased by the Purcell effect, but also by improving the emission extraction efficiency in PhC nanocavities^20^. On the other hand, we found that only one study has reported a small increase (70 percent) in the PL decay rate of Si nanocrystals embedded in a microdisk cavity although it is difficult to distinguish the radiative decay rate increase by the Purcell effect from the nonradiative decay increase induced by fabrications^22^. To obtain clear evidence of the Purcell effect, a simultaneous increase in the PL intensity and PL decay rate should be observed while nonradiative processes are carefully considered.

To demonstrate the Purcell effect on dopants in Si, we employed a Si PhC nanocavity doped with copper (Cu) atoms as radiative centres. Cu atoms in Si are known to form an isoelectronic centre (Cu-IEC), which is a deep impurity complex that binds an exciton^23^,^24^,^25^,^26^. The nonradiative Auger process in the Cu-IECs is prevented due to their neutral net charge. The narrow spectral linewidth of the Cu-IEC emission is suitable for obtaining an efficient spectral overlap with high-*Q* PhC nanocavities^27^. In this study, we observed large enhancements of the PL intensity and PL decay rate of the Cu-IECs in fabricated high-*Q* Si PhC nanocavities. We found that the rate enhancement depends on the *Q*/*V*_c_ values of the PhC cavities and the wavelength detuning between the cavity resonance and the Cu-IEC line. These experimental results exclude nonradiative contributions and provide clear evidence for the Purcell effect on Cu dopants in resonant Si PhC nanocavities for the first time.

## Results

**Figure 1:**
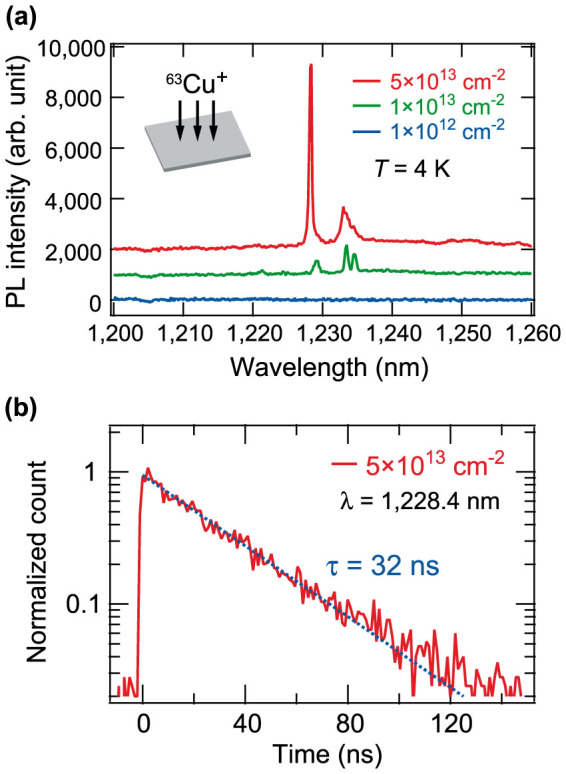
Photoluminescence of copper-doped Si films on SOI wafers. (a) PL spectra of the Si films with three different copper doses. A copper-related sharp emission is found at 1,228 nm. (b) PL decay of the emission at 1,228.4 nm with a bandwidth of 0.5 nm. The fitted exponential curve (broken line) provides a decay time of 32 ns. The sample temperature is 4 K.

First, we developed the fabrication of Si PhC cavities containing Cu-IECs. Figure 1(a) shows the PL spectra of Cu-doped thin Si films on silicon-on-insulator (SOI) wafers with three different Cu doses, which are platforms for Si PhC cavities. Although the Cu-IEC doping method with thermal Cu diffusion has already been reported for bulk Si substrates^23^,^24^,^25^,^26^,^27^, we adopted another way of doping thin Si films with Cu-IECs to fabricate PhC cavities in SOI wafers because the previous method does not control the dopant concentration precisely. We employed a combination of ion implantation and rapid thermal annealing. The doping method and the PL measurements are detailed in Supplementary Information and the Methods section, respectively. At a wavelength of 1,228.4 nm, an intense and narrow PL line is observed when the Cu ion dose is 5 × 10^13^ cm^−2^. The peak intensity of the line becomes larger as the Cu ion dose increases. This dose dependence indicates that the sharp PL line comes from Cu-related emission centres. With reference to previous studies^23^,^27^, we assigned the PL line at 1,228.4 nm as the zero-phonon line (ZPL) of the Cu-IECs because there is no other candidate for the radiative centre emitting photons around 1,228 nm. The small peaks seen at ~1,235 nm are the PL mediated by phonon emission. The ZPL has a small linewidth of 0.1 nm. These results show that our doping method successfully creates Cu-IECs in the thin Si films and enables us to fabricate Si PhC nanocavities containing the Cu-IECs. Figure 1(b) shows the time-resolved PL intensity of the ZPL for the sample with the highest dose. The exponential PL decay has a lifetime of 32 ns.

**Figure 2:**
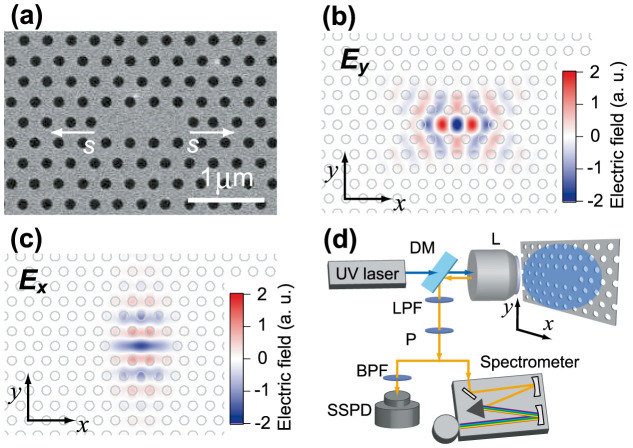
Structure of a copper-doped silicon photonic crystal cavity and the measurement set-up. (a) Scanning electron microscope image of an L3 cavity. The diameter of the air holes is 150 nm. The lattice period *a* is changed from 284 to 302 nm to coarsely tune the cavity resonance. The cavity *Q* is controlled by shifting the two end holes, denoted as *s*. Snapshot of the detected electric field amplitudes for (b) the fundamental and (c) second-order cavity modes, which were calculated by numerical simulations. The lowest *Q* cavity (*Q* = 450) is formed as the second-order mode of the L3 cavity. (d) Schematic of the measurement set-up. The sample is mounted on a cryostat. The laser irradiates the centre of the cavity with a spot diameter of ~2 μm. DM: dichroic mirror, L: objective lens, LPF: low-pass filter, P: polarizer, SSPD: superconducting single photon detector.

The air-suspended PhC membrane structures were fabricated on Si film doped with Cu at 5 × 10^13^ cm^−2^. Figure 2(a) shows a top-view image of the fabricated PhC cavity. The optical cavity was formed of three missing holes and is called an L3 cavity. Figure 2(b) and 2(c) show the electric field components of the fundamental and second-order cavity modes, which were calculated with the finite-difference time-domain (FDTD) method. The field of the fundamental (second-order) mode detected in our experiments is polarized perpendicular (parallel) to the long axis of the L3 cavity, indicated by *E_y_* (*E_x_*). We used the set-up shown in Fig. 2(d) to measure the PL of the fabricated Cu-doped PhC nanocavities. The sample temperature was 4 K in all the measurements.

**Figure 3:**
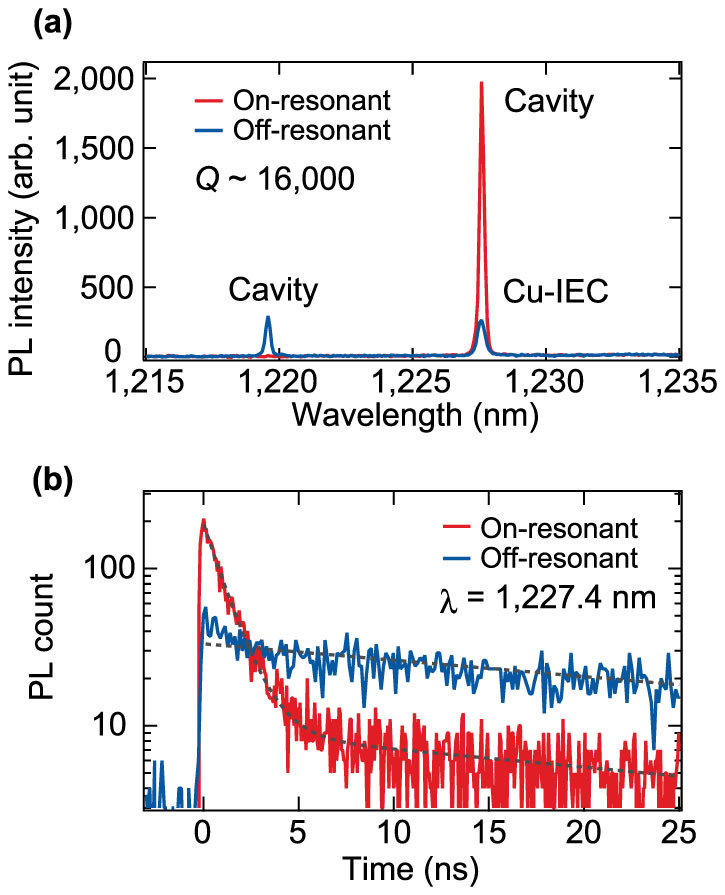
Photoluminescence of copper-doped silicon photonic crystal cavities. (a) PL spectra for the on- and off-resonant (detuning of −8 nm) cavities with the doped Cu-IECs. The *Q* factor of the cavity is ~16,000 with *s* = 0.10*a*. (b) PL decay of the Cu-IECs with the on- and off-resonant cavities. The fitted exponential curves shown as broken lines indicate decay lifetimes of 1.1 and 38 ns for the on-resonant cavity and 43 ns for the off-resonant cavity.

We studied the emission characteristics of the Cu-doped Si PhC cavities with PL measurements. Figure 3(a) shows the PL spectra of the PhC cavities with the highest *Q* of 16,000. It is clearly seen that the PL intensity of the ZPL of the Cu-IECs is greatly increased when the cavity is resonant with the Cu-IECs. The PL line of the off-resonant cavity possibly comes from background emissions from free electron-hole pairs or radiative defects^19^,^20^,^21^. Figure 3(b) highlights the PL decay of the Cu-IECs in the on- and off-resonant cavities. The PL decay time of the dominant component in the on-resonant state is 1.1 ns, which is noticeably shorter than the PL decay time for the unpatterned Cu-doped Si film (32 ns) as shown in Fig. 1(b). When the cavity resonance is detuned by −8 nm, the PL decay time of the Cu-IECs increases to 43 ns, which is longer than the decay time for the unpatterned Si film. This simultaneous enhancement of the PL intensity and PL decay rate suggests that the radiative process of the Cu-IECs is enhanced by the resonant Si PhC cavity. However, we cannot draw this conclusion only from Fig. 3 because the PL decay rate could be increased by the nonradiative recombination centres unintentionally produced during sample fabrication, and also because the intensity could be increased by the large emission extraction efficiency of the PhC cavity. To confirm the enhancement of the radiative recombination of the Cu-IECs, next we systematically investigated the PL intensity of the Cu-IECs and its decay rate as we changed the wavelength detuning between the Cu-IEC line and the cavity resonance, and the cavity *Q*/*V*_c_ value.

**Figure 4:**
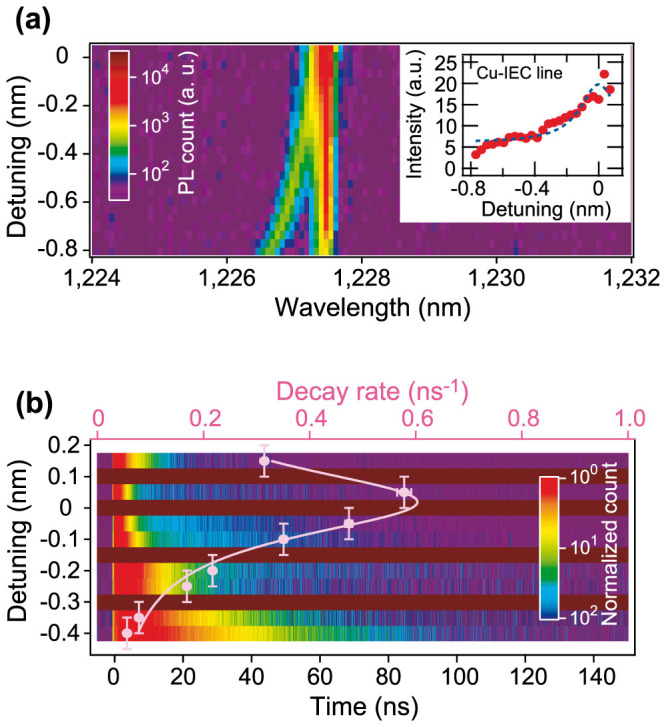
Detuning dependence of photoluminescence for copper-doped nanocavities. (a) PL spectrum map as a function of the spectral detuning between the cavity and the Cu-IEC line. The inset shows the integrated PL intensity of the Cu-IEC line with a Lorentz curve with a spectral width of 0.28 nm. (b) PL decay and decay rates of the Cu-IEC line depending on the detuning. The detuning error bars indicate the bandwidth of the optical filter (0.1 nm). A Lorentz curve with a spectral width of 0.28 nm is well fitted to the data.

Figure 4(a) and 4(b) show density plots of PL spectra and PL decay as a function of cavity detuning from the Cu-IEC line. The fine cavity tuning was accomplished by depositing xenon on the sample^28^. Here we measured another cavity with *Q* = 7,200. This broader cavity linewidth makes it easier to adjust the cavity resonance to the ZPL of the Cu-IECs. The cavity *Q* value was not changed during the tuning. Figure 4(a) clearly indicates that as the cavity resonance approaches the Cu-IEC line, the PL peak intensity of the Cu-IEC line is gradually increasing. To see this more precisely, we plotted the integrated PL intensity of the Cu-IECs in the inset of Fig. 4(a). The integrated intensity at zero detuning is 6 times larger than that at a detuning of −0.8 nm. The density plot in Fig. 4(b) shows that the PL decay of the Cu-IECs becomes faster as the detuning becomes smaller. We plot the decay rate extracted from exponential fittings to the measured PL decay data. At almost zero detuning, the PL decay rate is 0.58 ns^−1^, which is 10 times larger than the rate of 0.06 ns^−1^ at a detuning of −0.4 nm. It is worth noting that the detuning dependence of the PL decay rate is well fitted by the Lorentz curve with a spectral linewidth of 0.28 nm which is comparable to the cavity linewidth of 0.17 nm at *Q* = 7,200. The Lorentz curve with the same linewidth also fits the detuning dependence of the integrated PL intensity as seen in the inset of Fig. 4(a).

The experimental results in Fig. 4 show that both the PL intensity and PL decay rate of the Cu-IECs clearly exhibit a Lorentzian dependence on the detuning between the cavity resonance and the Cu-IEC line. Since nonradiative decay is independent of the cavity resonance, this detuning dependence excludes the increase in the PL decay rate by nonradiative processes. In addition, we found that the PL intensity enhancement is positively correlated with the PL decay rate enhancement. This finding also excludes nonradiative contributions to the PL decay rate enhancement because nonradiative decay should result in a negative correlation between the PL intensity and PL decay rate.

**Figure 5:**
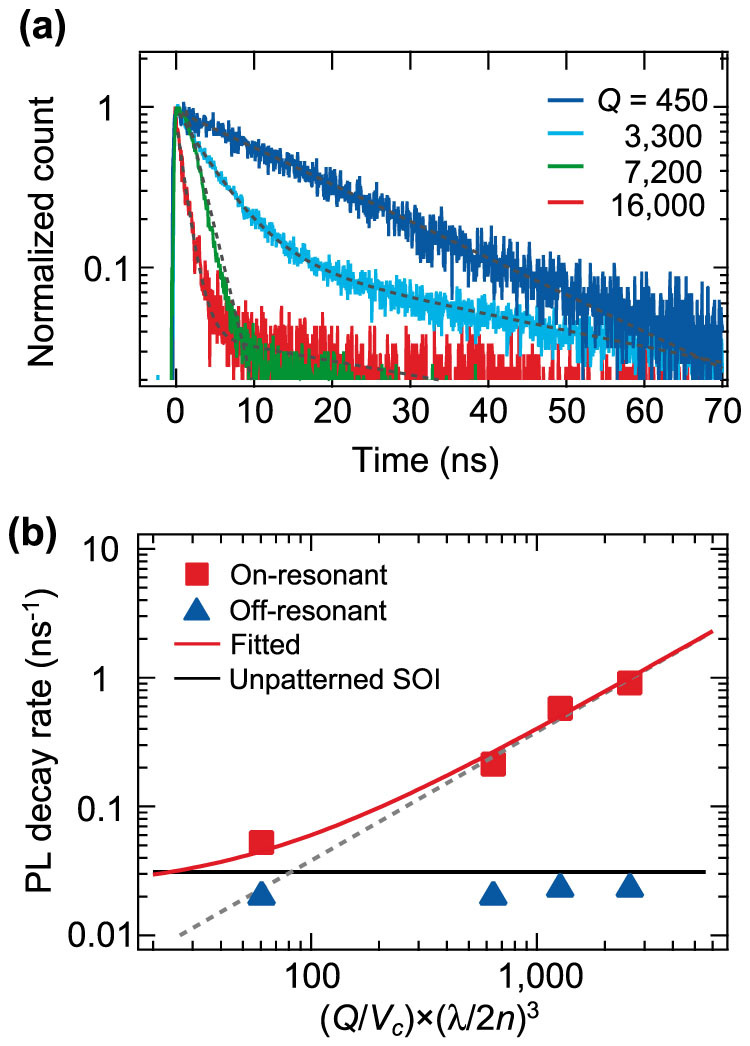
Cavity *Q*/*V*_c_ dependence of photoluminescence for copper-doped nanocavities. (a) PL decay curves of the Cu-IECs in the on-resonant cavities with different *Q* values. The broken lines indicate the fitted exponential curves. (b) PL decay rates of the Cu-IECs in the on- and off-resonant cavities as a function of the normalized *Q*/*V*_c_ values. The value of (*Q*/*V*_c_) × (*λ*_c_/2*n*)^3^ on the horizontal axis is an approximate measure of the theoretical Purcell factor. The broken line fitted to the on-resonant data is an eye guide without a *Q*/*V*_c_-independent nonradiative decay rate. The solid red curve is the fitted curve considering the nonradiative decay rate of 0.022 ns^−1^. The horizontal black line indicates the PL decay rate of the Cu-IECs in the unpatterned Si film.

If the observed cavity enhancements are due to the Purcell effect on the Cu-IECs, we should be able to observe the PL decay rate, which is proportional to the *Q*/*V*_c_ value of the PhC cavity^14^. This *Q*/*V*_c_ dependence is the clearest proof of the Purcell effect. In our experiments, we examined different samples with cavity *Q* values ranging from 450 to 16,000. The *Q* value was changed by shifting the position of the end holes of the L3 cavity without greatly changing *V*_c_^29^. The cavity parameters are shown in Table 1. In Fig. 5(a), we plot the PL decay curves for the Cu-IECs doped in the on-resonant cavities with four different *Q* values. These four curves show that the PL decay is accelerated as the cavity *Q* increases. The longest decay time is 19 ns for the lowest *Q* cavity (*Q* = 450), and the shortest decay time is 1.1 ns for the highest *Q* cavity (*Q* ~ 16,000). In fact, when changing the cavity *Q*, the mode volume changes slightly as shown in Table 1. Thus, the relevant figure is not *Q* itself, but *Q*/*V*_c_. Figure 5(b) shows the PL decay rate as a function of *Q*/*V*_c_. This figure shows that the PL decay rate for the on-resonant cavity is proportional to *Q*/*V*_c_. As an eye guide, we performed a linear fitting while neglecting the *Q*/*V*_c_-independent nonradiative decay rate. The fitted broken line in Fig. 5(b) agrees with the data points except for that of the lowest *Q*/*V*_c_. This linear relationship between the PL decay rate and the *Q*/*V*_c_ value is clear and conclusive evidence that the PL decay rate is indeed increased by the Purcell effect on the Cu-IECs. Although the PL intensity enhancement has considerable uncertainty due to the experimental inaccuracy of the detection efficiency for emission, the PL rate enhancement observed in the time-resolved measurements is sufficiently accurate to prove the Purcell effect because it is not affected by the detection efficiency.

Figure 5(b) shows the PL decay rates for the unpatterned Si film and the off-resonant cavities. At the highest *Q*/*V*_c_ value, the PL decay rate for the on-resonant cavity is 30 times larger than the rate for the unpatterned Si film. In contrast, the PL decay rates for the off-resonant cavities show no noticeable dependence on *Q*/*V*_c_. Their decay rates are slightly smaller than the rate for the unpatterned Cu-doped Si film.

We obtained two key results in our experiments. First, the PL measurements performed while tuning the cavity resonance revealed that the PL intensity and PL decay rate of the Cu-IECs are resonantly increased by the Si PhC nanocavities, not by nonradiative processes induced by sample fabrication. The second key result is that the PL decay rate is in proportion to the cavity *Q*/*V*_c_, which is derived from the PL decay measurements of Si PhC cavities with different *Q* values. These results constitute the first unambiguous experimental demonstration of the Purcell effect on dopants in Si. In the next section, these results are used for a quantitative discussion of the Purcell enhancement and the intrinsic radiative rate of Cu-IECs.

## Discussion

Based on the experimental results, we discussed the relationship between the PL intensity enhancement and the PL decay rate enhancement with a model designed to estimate the radiative decay rate enhancement in on-resonant cavities (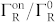) and its suppression in off-resonant cavities (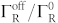). Here, 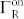, 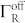, and 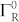 are the radiative decay rates of the Cu-IECs in the on- and off-resonant cavities, and its intrinsic radiative decay in the unpatterned Cu-doped Si film, respectively. The estimation procedures based on experimental results are described in detail in Supplementary Information. At the highest *Q* of 16,000, 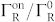 is estimated to be 144, which is approximately 1/10 of the theoretical Purcell factor (*F_p_*) shown in Table 1. This difference in the radiative rate enhancement, which is described by a factor 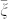 ~ 0.1, results from the spatial and polarization mismatches between the cavity-confined electric fields and the emission dipole moments of the Cu-IECs distributed in the cavity^30^. The polarization coupling factor is estimated to be ~0.3 (see Supplementary Information), indicating that the spatial coupling factor might be ~0.3. In addition, the 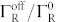 value estimated for the off-resonant cavities is about 1/30. This radiative decay rate reduction is due to the suppression of spontaneous emission by the photonic bandgap^31^,^32^, which causes the slight difference between the PL decay rates of the off-resonant cavities and the unpatterned Si film as seen in Fig. 5(b).

From the 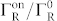 and 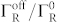 values, the unknown nonradiative decay rate 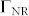 and 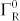 values are also estimated to be 0.020 ~ 0.023 ns^−1^ and 2 ~ 22 μs^−1^, respectively. The estimation procedure is detailed in Supplementary Information. Using the average 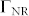 of 0.022 ns^−1^, in Fig. 5(b) we plot a red solid curve assuming that the PL decay rate for the on-resonant cavity is described by 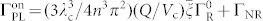, where *λ*_c_ is the cavity resonant wavelength, *n* is the refractive index of Si, and 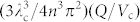 is the theoretical Purcell factor. The curve is well fitted to all the measured data. This result indicates that the deviation from the linear fitting (shown by a grey broken line) at a low *Q*/*V*_c_ is due to the nonradiative decay rate. Furthermore, we found that the estimated 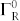 (2 ~ 22 μs^−1^) is about three orders of magnitude larger than the rate of about 0.01 μs^−1^ that was previously reported for the Cu-IECs in bulk Si fabricated by thermal Cu diffusion^23^,^25^. The 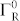 value estimated from 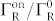 and 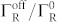 has a large variation because this estimation uses a PL intensity enhancement that contains a large uncertainty due to the experimental set-up. To confirm the difference in 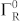, we examine the 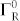 value using another approach. In the curve fitted to the data in Fig. 5(b), we estimated 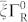 ~ 0.6 μs^−1^ from the slope. Since the coupling factor 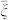 is less than unity, 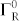 must be limited by the lower bound rate of 0.6 μs^−1^, which is still two orders of magnitude larger than previously reported. Thus, the difference between our estimated 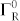 and the reported values is significant. Although further studies are necessary to understand this difference, one possible explanation for the discrepancy is related to the Cu doping procedure. Our ion implantation is able to create Cu-IECs in a thin Si film with a higher concentration than with the previous Cu diffusion method. The radiative decay of these dense Cu-IECs could be accelerated by the superradiance effect in a thin Si film, which takes place in an emitter ensemble localized in a sub-wavelength-sized volume with little spectral broadening^33^,^34^.

## Conclusion

The present study demonstrated large increases in the PL intensity and PL decay rate of Cu-IECs achieved by using Si PhC nanocavities, comparing with an unpatterned Cu-doped Si film. We found that the PL decay rate exhibited Lorentzian behaviour as regards the cavity detuning, and the PL decay rate was linearly proportional to the *Q*/*V*_c_ of the on-resonant cavity. These experimental results excluded the possibility of nonradiative decay contributing to the PL decay rate enhancement, and they proved unambiguously that the spontaneous emission rate of the Cu-IECs is indeed increased by the cavity resonance, that is, the Purcell effect. In a cavity with *Q* ~ 16,000, the Cu-IECs showed ultrafast PL decay with a rate of ~1 ns^−1^, which is 30 times larger than the PL decay rate for the reference sample. This ultrafast radiative decay is as fast as the intrinsic radiative decay of the III/V semiconductor quantum dots^35^,^36^, which is in an unprecedented regime for light emission in Si.

It is theoretically predicted that the Purcell enhancement is limited by the finite line broadening of the emitter^15^. Previous studies showed that we can obtain an ultra-narrow Cu-IEC emission with a linewidth of 4.6 pm by employing isotope-purified Si^27^. Since we are able to fabricate ultrahigh-*Q* PhC cavities with *Q* > 10^6^ 37, the combination of this ultra-narrow emission of the Cu-IECs and ultrahigh-*Q* cavities may realize a larger enhancement of the Cu-IEC emission.

Our results demonstrating the Purcell effect on Cu dopants in Si PhC nanocavities indicate a cavity-enhanced light-dopant interaction that is capable of improving emission characteristics and optical access for dopants in Si. We suggest that the high-*Q* Si PhC nanocavity coupled with dopants may be a promising component for realizing bright Si light emitting devices and Si quantum optical devices based on dopants with efficient optical access similar to those demonstrated in diamond^38^,^39^.

## Methods

For our low-temperature PL measurements, we used the set-up shown in Fig. 2(d). The samples were cooled to 4 K by a helium flow cryostat. A 50× objective with a numerical aperture (NA) of 0.42 focused a continuous ultraviolet (UV) laser on the centre of the cavity with a spot diameter of ~2 μm. The laser wavelength was 375 nm and the average incident power was 420 μW. At this wavelength, the incident power was mostly absorbed by the 300-nm-thick Si layer. The same objective simultaneously collected the PL from the excited area of the samples. The PL was guided by an optical fibre to a spectrometer attached to an InGaAs detector array. The spectral resolution was ~0.07 nm. A polarizer was placed in front of the spectrometer to detect the *E_y_* or *E_x_* component of the Cu-IEC emission coupling to the cavity mode. To measure the time-resolved PL, the samples were excited by using the pulsed UV laser with a pulse width of 0.5 ns, and a repetition rate of 5 MHz. The average power was 18 μW. The PL was spectrally filtered by a band pass filter at the wavelength of interest with a bandwidth of 0.1 or 0.5 nm. The filtered emission was detected by a superconducting single photon detector (SSPD). The photon-counting signal from the SSPD was fed to a time-to-amplitude converter triggered by the laser, and then the PL decay curve was recorded. The time resolution of this measurement system was ~50 ps.

## Author Contributions

H.S. planned the project, performed the sample design, optical experiments, and data analyses, and wrote the manuscript. E.K. and H.T. supported the sample fabrication and the FDTD simulations, respectively. M.N. led the project and wrote the manuscript. All the authors contributed to the manuscript.

## Supplementary Material

Supplementary Information Ultrafast spontaneous emission of copper-doped silicon enhanced by an optical nanocavity

## Figures and Tables

**Table 1:** Characteristic parameters of on- and off-resonant cavities, and an unpatterned Si film. The quality factor (*Q*), volume (*V*), and theoretical Purcell factor (*F_p_*) of the cavities are calculated by FDTD simulations. The experimental *Q* values estimated from the PL spectra are almost as same as the simulated *Q* values. *s* and *a* are the position shift of the end holes and lattice period of the cavities, respectively. 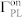 and 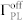 are the observed PL decay rates of the Cu-IECs in the on- and off-resonant cavities, respectively

*s*	*Q*	*V* (μm^3^)	*F_p_*	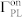 (ns^−1^)	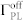 (ns^−1^)
0.08a*	450	0.042	33	0.053	0.020
−0.07*a*	3,300	0.029	350	0.21	0.020
0.03*a*	7,200	0.032	690	0.58	0.023
0.10*a*	16,000	0.035	1,400	0.91	0.023
Off-resonant cavity	-	0.73†	-	-	0.022‡
Unpatterned	-	0.94†	-	0.031	

*This results from the second-order mode of the L3 cavity. The others result from the fundamental mode.

^†^The volume is equal to the Si volume excited by the laser with a spot diameter of ~2 μm.

^‡^The PL decay rate is the average rate observed in the off-resonant cavities.
