# Supplementary material for: Ultrafast spontaneous emission of copper-doped silicon enhanced by an optical nanocavity
Source: Sci Rep. 2014 May 23;4:5040. doi: 10.1038/srep05040 (PMC4031467; doi:10.1038/srep05040)
Supplement: Supplementary Information — Ultrafast spontaneous emission of copper-doped silicon enhanced by an optical nanocavity [file srep05040-s1.doc]

**Supplementary Information**

**Ultrafast spontaneous emission of copper-doped silicon enhanced by an optical nanocavity**

HISASHI SUMIKURA^1,2^*, EIICHI KURAMOCHI^1,2^, HIDEAKI TANIYAMA^1,2^ AND MASAYA NOTOMI^1,2^

^1^NTT Basic Research Laboratories, NTT Corporation, 3-1 Morinosato Wakamiya, Atsugi, Kanagawa 243-0198, Japan

^2^NTT Nanophotonics Center, NTT Corporation, 3-1 Morinosato Wakamiya, Atsugi, Kanagawa 243-0198, Japan

*e-mail: sumikura.hisashi@lab.ntt.co.jp

**1. Copper doping method**

In previous reports, thick Si substrates are doped with copper isoelectronic centres (Cu-IECs) in the following way. First, a Cu film is deposited on a Si substrate, and annealed to diffuse Cu atoms into the Si. The annealed substrate is then dropped into cold liquid for rapid thermal quenching [S1]. This thermal quenching process is crucial for forming Cu-IECs. However, since this method does not allow precise control of the doping conditions, we have developed another method for doping a thin Si film on a silicon-on-insulator (SOI) wafer, which is a photonic crystal (PhC) platform, with Cu-IECs. Our method employs ion implantation and rapid thermal annealing (RTA). The ion implantation makes it possible to precisely control the doping concentration, position, depth, and isotopes. The RTA can cool samples rapidly in a well-controlled way.

The SOI wafer used in our study consists of an undoped 300-nm-thick Si film on a 2-μm-thick buried oxide layer. ^63^Cu ions were implanted over the entire top surface of the Si film with an acceleration voltage of 100 kV. The Cu ions were broadly distributed in the thin Si film and the Cu density peaked at a depth of 80 nm. To form Cu-IECs, the Cu-implanted SOI wafer was annealed by an RTA machine for 30 seconds at 800 ºC. The cooling speed was around 50 K/s. Approximately 50,000 Cu atoms are implanted in a PhC nanocavity when the ion dose is 5×10^13^ cm^-2^.

In photoluminescence (PL) measurements for the Cu-doped Si films, an ultraviolet excitation laser creates free electron-hole (*e*-*h*) pairs in the Si films with a concentration of ~4×10^17^ cm^-3^. These dense free *e*-*h* pairs disappear within 0.5 ns due to fast *e*-*h* capture by Cu impurities and nonradiative surface recombination [S2]. This was experimentally confirmed by the quick rise of the time-resolved PL of the Cu-IECs under pulse laser excitation as seen in Fig. 1(b). An *e*-*h* pair captured by a Cu impurity complex forms a Cu-IEC and then emits a photon with radiative *e*-*h* recombination. In PL measurements at 4 K, we found that the zero phonon line of the doped Cu-IECs has a narrow linewidth. The linewidth of 0.1 nm was maintained as the Cu dose increased. This indicates that the individual Cu-IEC is well isolated and there is no impurity band formed by the Cu-IECs. The PL lines were slightly shifted to a shorter wavelength by the higher ion dose. This is possibly due to the change of the strain in the Cu-doped Si film on the SOI wafer, which is induced by ion implantation. As the sample temperature elevated to 50 K, the Cu-IEC line became broader and weaker. At room temperature, the line disappeared completely.

**2. Photoluminescence intensity ratio**

The PL intensity ratio *K* is defined as the PL intensity of Cu-IECs in a nanocavity, which is divided by their intensity in an unpatterned Si film. The *K* values for on- and off-resonant cavities are respectively described by

$K_{\text{on}}=I_{\text{on}}/I_{0}$ and $K_{\text{off}}=I_{\text{off}}/I_{0}$, (1)

where *I*_on_, *I*_off_, and *I*_0_ are the integrated PL intensities of the Cu-IECs in on- and off-resonant cavities and an unpatterned Cu-doped Si film, respectively. Here we mention an estimation approach for calculating *I*_on_. In our experiments, the excitation and detection areas are larger than the PhC cavities. Thus, the PL detected by our set-up inevitably includes emissions from both the cavity and the outside of the cavity excited by the laser. The cavity is surrounded by the PhC structure forming the photonic bandgap (PBG), which is equivalent to the case with Cu-IECs in off-resonant cavities. To estimate the Cu-IEC emission only from the resonant cavity, we subtracted *I*_off_ from the measured PL intensity for the cavity *I*_meas_ as described by *I*_on_ = *I*_meas_ - *I*_off_. In fact as shown in Fig. 3(b), the on-resonant PL decay includes a slow decay component with a PL lifetime comparable to the off-resonant case. This slow component may result from the Cu-IECs in the PhC structure outside the cavity. In contrast, since the emitter volumes for *I*_off_ and *I*_0_ are equal to the excitation volume determined by the spot size of the focused excitation laser, no correction for calculating *K*_off_ is necessary in the off-resonant case.

*K*_on_ and *K*_off_ values for every cavity estimated from the PL intensities are summarized in Table S1. *K*_on_ is always larger than *K*_off_ by a factor of less than 10 although the *K*_on_ and *K*_off_ values are rather scattered. The scattering of the *K*_on_ values arises from the considerable variation in the PL intensities. This is because the measured PL intensities for small nanocavities are fluctuating by slight positional misalignments of the small excitation and detection spot in our confocal micro-PL set-up.

**3. Emission quantum efficiency ratio**

We examined the relationship between PL intensity and PL decay rate enhancements. The PL intensity is proportional to *ηβV*, where *η* is the internal quantum efficiency (QE) for emission, *β* is the extraction and detection efficiency of light emitted from a Si sample, and *V* is the volume of the excited emitters. Thus, the following relations for *K*_on_ and *K*_off_ are satisfied,

$K_{\text{on}}=\frac{\eta_{\text{on}}\beta_{\text{on}}V_{\text{c}}}{\eta_{0}\beta_{0}V_{0}}$ and $K_{\text{off}}=\frac{\eta_{\text{off}}\beta_{\text{off}}V_{\text{off}}}{\eta_{0}\beta_{0}V_{0}}$. (2)

We estimated *β* with finite-difference time-domain (FDTD) simulations calculating the Poynting vector directed to the objective [S3], and *V* was determined from the cavity mode size and the diameter of the excitation laser spot, as summarized in Table S1. Equation (2) and experimental *K* values enabled us to obtain the QE ratios expressed by *η*_on_ /*η*_0_ and *η*_off_ /*η*_0_ for the on- and off-resonant cavities, respectively. These results are shown in Fig. S1(a). *η*_on_ /*η*_0_ and *η*_off_ /*η*_0_ are scattered in the cavity-*Q*/*V*_c_ dependence. This is because of experimental errors for the *K* values estimated from the measured PL intensities.

**4. Radiative decay rate ratio**

We estimated the radiative decay rate ratios, $\Gamma_{\text{R}}^{\text{on}}/\Gamma_{\text{R}}^{0}$ and $\Gamma_{\text{R}}^{\text{off}}/\Gamma_{\text{R}}^{0}$ for on- and off-resonant cavities without using the Purcell theory, where $\Gamma_{\text{R}}^{\text{on}}$ and $\Gamma_{\text{R}}^{\text{off}}$ are the radiative decay rates of the Cu-IECs in on- and off-resonant cavities, respectively. $\Gamma_{\text{R}}^{0}$ is the intrinsic radiative rate of the Cu-IECs in an unpatterned Si film. The PL decay rate obtained by time-resolved PL measurements is described by Γ_PL_ = Γ_R_ + Γ_NR_, where  Γ_NR_ is the nonradiative recombination rate. Since the emission QE is given by $\eta=\Gamma_{\text{R}}/(\Gamma_{\text{R}}+\Gamma_{\text{NR}})$, the relations,

$\frac{\Gamma_{\text{R}}^{\text{on}}}{\Gamma_{\text{R}}^{0}}=\frac{\eta_{\text{on}}}{\eta_{0}}\frac{\Gamma_{\text{PL}}^{\text{on}}}{\Gamma_{\text{PL}}^{0}}$ and $\frac{\Gamma_{\text{R}}^{\text{off}}}{\Gamma_{\text{R}}^{0}}=\frac{\eta_{\text{off}}}{\eta_{0}}\frac{\Gamma_{\text{PL}}^{\text{off}}}{\Gamma_{\text{PL}}^{0}}$ (3)

are obtained. Since the QE and the PL decay rate ratios were respectively given by equation (2) and time-resolved PL measurements, $\Gamma_{\text{R}}^{\text{on}}/\Gamma_{\text{R}}^{0}$ and $\Gamma_{\text{R}}^{\text{off}}/\Gamma_{\text{R}}^{0}$ were estimated only from the experimental PL intensity and its decay data without any assumptions. The results are shown in Fig. S1(b). Although the PL decay rate is precisely measured, the scattered QE ratios increase the errors for the radiative decay rate ratios. In the on-resonant cavities, $\Gamma_{\text{R}}^{\text{on}}$ is increased in proportion to *Q/V*_c_, but *η*_on_ is not. The largest rate enhancement $\Gamma_{\text{R}}^{\text{on}}/\Gamma_{\text{R}}^{0}$ is ~144 at *Q* ~ 16,000. Since the intrinsic radiative rate of the Cu-IECs is small, we can observe a large radiative rate enhancement without entering the strong coupling regime [S4].

For the off-resonant case, we clearly found a reduction in the QE and the radiative decay rate. *η*_off_ and $\Gamma_{\text{R}}^{\text{off}}$are more than an order of magnitude smaller than *η*_0_ and $\Gamma_{\text{R}}^{0}$, respectively. We believe that these suppressions result from the inhibition of spontaneous emission due to the PBG. The suppression ratio of $\Gamma_{\text{R}}^{\text{off}}/\Gamma_{\text{R}}^{0}$ ~ 1/30 is comparable to an experimental result obtained for quantum dots embedded in the PBG of two-dimensional PhCs [S5].

**5. Intrinsic radiative decay rate and nonradiative decay rate**

In this section, we show a way of estimating the intrinsic radiative decay rate and the nonradiative decay rate of the Cu-IECs in every cavity. We assumed that the nonradiative decay rate is independent of the detuning between the cavity resonance and the Cu-IEC line. The PL decay rates for on- and off-resonant cavities, which are respectively described by $\Gamma_{\text{PL}}^{\text{on}}=\Gamma_{\text{R}}^{\text{on}}+\Gamma_{\text{NR}}$ and $\Gamma_{\text{PL}}^{\text{off}}=\Gamma_{\text{R}}^{\text{off}}+\Gamma_{\text{NR}}$, derive the relation of

$\Gamma_{\text{R}}^{\text{on}}-\Gamma_{\text{R}}^{\text{off}}=\Gamma_{\text{PL}}^{\text{on}}-\Gamma_{\text{PL}}^{\text{off}}$. (4)

On the other hand, when the radiative decay rate ratios obtained by equation (3) are expressed by $\alpha_{\text{on}}$ and $\alpha_{\text{off}}$, equation (4) is reformed to

$\Gamma_{\text{R}}^{0}=(\Gamma_{\text{PL}}^{\text{on}}-\Gamma_{\text{PL}}^{\text{off}})/(\alpha_{\text{on}}-\alpha_{\text{off}})$. (5)

In this equation, $\Gamma_{\text{PL}}^{\text{on}}-\Gamma_{\text{PL}}^{\text{off}}$ was experimentally obtained in the PL decay measurements, and $\alpha_{\text{on}}-\alpha_{\text{off}}$ was estimated from the experimental results shown in Fig. S1(b). By equation (5), the nonradiative decay rate is estimated from the relation,

$\Gamma_{\text{NR}}=\Gamma_{\text{PL}}^{\text{on}}-\alpha_{\text{on}}\Gamma_{\text{R}}^{0}$ or $\Gamma_{\text{NR}}=\Gamma_{\text{PL}}^{\text{off}}-\alpha_{\text{off}}\Gamma_{\text{R}}^{0}$. (6)

The estimated intrinsic radiative decay rates and nonradiative decay rates are summarized in Table S1. Since these calculations are based on the scattered PL intensity enhancement ratios *K*_on_, the $\Gamma_{\text{R}}^{0}$ error is large, which is seen from the large variation of $\Gamma_{\text{R}}^{0}$ in Table S1.

In contrast, the estimated nonradiative decay rates are in the 0.020 to 0.023 ns^-1^ range, and there is no significant dependence on the cavities. This indicates that the nonradiative decay of the Cu-IECs is not changed by the fabrication of air hole arrays in PhC structures. Since a Cu-IEC strongly binds an exciton around the Cu atoms thus preventing the bound exciton from being diffused, the Cu-IEC far from the air holes is insensitive to the nonradiative surface recombination at the air holes. The nonradiative decay possibly originates from defects and dislocations in the Cu-doped Si films, which are created by ion implantation. In fact, we found point defects and dislocations in transmission electron microscope pictures.

**6. Purcell effect on distributed Cu-IECs**

We discuss the Purcell effect on the Cu-IECs distributed in the PhC cavity and compare the experimental results with the Purcell theory. In an optical cavity, the radiative decay rate of a single emitter at an emission frequency *ω*_a_ is described by

(7)

where *d*_0_, *n*, *V*_c_, *ω*_c_, and *κ* are the emission dipole strength, the refractive index of the material, the mode volume of the cavity, the cavity resonance, and the cavity damping rate, respectively [S6]. *κ* is derived from *ω*_c_/*Q*. The coupling factor *ξ*(***r***) is defined by $|\mathbf{E}(\mathbf{r})\cdot\mathbf{e}|^{2}/\max(|\mathbf{E}|^{2})$, which takes account of the electromagnetic coupling strength at a position ***r*** between the optical electric field ***E*** in the cavity and the dipole oriented along a unit vector ***e***. In equation (7), we assumed that the homogeneous linewidth of the emitter is sufficiently narrower than the cavity linewidth. This assumption is reasonable in our case. The linewidth of the Cu-IECs observed in our natural Si samples was 0.1 nm. However, it is reported that the Cu-IECs in isotope-purified Si exhibit a very narrow linewidth in high-resolution PL measurements, which is around 4.6 pm [S7]. This means that the PL linewidth in our samples is broadened by the inhomogeneity of the Si isotope impurity found in natural Si because the homogenous linewidth of a single Cu-IEC should be less than 4.6 pm at least. This homogeneous linewidth is approximately 1/15 of the cavity linewidth of ~0.07 nm for the highest *Q* cavity.

In our samples, the Cu-IECs were dispersed throughout entire Si films, and many Cu-IECs could be found in the PhC cavity. The Cu-IECs have different radiative decay rates depending on their position and their dipole orientation. The observed radiative decay of the Cu-IECs should be the sum of the decay of individual Cu-IECs because our micro-PL measurements cannot resolve emissions from individual Cu-IECs. If we assume that the individual emitters are completely independent and there is no interaction among them, the time evolution of the PL from the emitter ensemble in a resonant cavity is expressed by

$G_{\text{PL}}^{\text{on}}(t)=N\int_{V_{\text{m}}} d\mathbf{r}\int_{0}^{\pi} d\theta\int_{0}^{\pi} d\phi\int_{-\infty}^{\infty} d\omega_{\text{a}} g(\omega_{0})\gamma_{\text{R}}^{\text{on}}\exp[-(\gamma_{\text{R}}^{\text{on}}+\gamma_{\text{NR}})t]$, (6)

where *N*, *V*_m_, and *γ*_NR_ are the number of the emitters, the detection volume, and the nonradiative decay rate of an emitter. The inhomogeneous broadening of the emitters is expressed by a normalized distribution function *g*(*ω*_0_) with a centre frequency *ω*_0_. In addition, *ξ*(***r***) is reformed to $(E(\mathbf{r})\sin\phi\cos\theta)^{2}/\max(E^{2})$, where *E*(***r***), *θ,* and *ϕ* are the electric field amplitude at a position ***r***, and the polar and azimuth angles of the dipole, respectively. Although the dipole is randomly oriented, its emission is dominated by the fast emission polarized along the cavity-confined electric field. The numerical calculation for equation (6) shows that the PL decay is no longer mono-exponential decay, but stretched exponential decay [S8-S10].

However, our time-resolved PL measurements revealed mono-exponential or double exponential PL decay for the Cu-IECs. We found that the exponential PL decay we observed cannot be reproduced by the stretched exponential decay calculated by equation (6). This disagreement suggests that the assumption for this calculation is not applicable to our case and a different assumption is needed.

Next, we assumed that the emitters interact with each other. In this case, since the emitter ensemble can be assumed to be a single emitter, the PL decay for the cavity shows mono-exponential decay with the averaged radiative decay rate described by

$\Gamma_{\text{PL}}^{\text{on}}=\int_{V_{\text{m}}} d\mathbf{r}\int_{0}^{\pi} d\theta\int_{0}^{\pi} d\phi\int_{-\infty}^{\infty} d\omega_{\text{a}} g(\omega_{0})\gamma_{\text{R}}^{\text{on}}(\omega_{0},\mathbf{r},\theta,\phi)$. (7)

When *g*(*ω*_0_) is a normalized Gaussian function with a linewidth of Δ*ω*_0_ and $\delta\sim\sqrt{\kappa^{2}+\Delta\omega_{0}^{2}}$ is defined, equation (7) is approximated to

. (8)

$\bar{\xi}$ is the spatial and polarization coupling factor averaged over all the emitters in the cavity, which includes a polarization coupling factor of ~0.3. This polarization coupling factor is obtained by the fact that the single polarization parallel to the resonant cavity field contributes to the cavity-enhanced Cu-IEC emission although the degree of freedom in the polarization of the emission is 3. Δ*ω*_0_ means the inhomogeneous broadening of the emitters. The detuning dependence of the measured PL decay rate in Fig. 4(b) is explained by equation (8). In the present case, the inhomogeneous broadening is 0.1 nm, and the cavity linewidth is 0.17 nm at *Q* = 7,200. Taking account of the instrumental spectral resolution *σ* of 0.1 nm, the spectral linewidth for $\Gamma_{\text{PL}}^{\text{on}}$ is theoretically estimated by $\sqrt{\delta^{2}+\sigma^{2}}$~ 0.22 nm, which is comparable to the measured linewidth of 0.28 nm. This indicates that the experimental result is in good agreement with our Purcell theory including the inhomogeneous broadening.

When $\kappa>\Delta\omega_{0}$ in low *Q* cavities, equation (8) at zero detuning is expressed by

$\Gamma_{\text{R}}^{\text{on}}\approx F_{p}\bar{\xi}\Gamma_{\text{R}}^{0}$, (9)

where the theoretical Purcell factor *F_p_* and the intrinsic radiative decay rate $\Gamma_{\text{R}}^{0}$ are respectively defined by [S11]

$F_{p}=\frac{6\pi c^{3}}{n^{3}\omega_{\text{c}}^{3}}\frac{Q}{V_{\text{c}}}$ and . (10)

When we take the nonradiative decay rate of the Cu-IEC ensemble into account, the PL decay rate in a resonant cavity is given by

$\Gamma_{\text{PL}}^{\text{on}}\approx F_{p}\bar{\xi}\Gamma_{\text{R}}^{0}+\Gamma_{\text{NR}}$. (11)

Based on the Purcell theory, we can estimate the coupling factor $\bar{\xi}$ by fitting equation (9) to Fig. S1(b). This line fitting led to $\bar{\xi}$ = 0.10 ± 0.03. Using $F_{p}\bar{\xi}(\approx\Gamma_{\text{R}}^{\text{on}}/\Gamma_{\text{R}}^{0})$ in equation (9) and experimental $\Gamma_{\text{PL}}^{0}$ and $\Gamma_{\text{PL}}^{\text{on}}$ values, we derived *η*_on_/*η*_0_ with equation (3). The theoretical curve for *η*_on_/*η*_0_ is shown in Fig. S1(a). Note that it is now clear that *η*_on_/*η*_0_ is saturated when *Q*/*V*_c_ is larger than 100(λ/2*n*)^3^. When the radiative decay rate becomes larger than the nonradiative decay rate as a result of the cavity enhancement at higher *Q*/*V*_c_ values, QE is saturated at unity and is less dependent on *Q*/*V*_c_. At the highest *Q*/*V*_c_, the emission QE is estimated to be 0.98 from the relation, $\eta_{\text{on}}=\Gamma_{\text{R}}^{\text{on}}/(\Gamma_{\text{R}}^{\text{on}}+\Gamma_{\text{NR}})=(\Gamma_{\text{PL}}^{\text{on}}-\Gamma_{\text{NR}})/\Gamma_{\text{PL}}^{\text{on}}$, where $\Gamma_{\text{PL}}^{\text{on}}$ = 0.91 ns^-1^ and the average Γ_NR_ = 0.022 ns^-1^ as listed in Table S1.

In addition, the intrinsic radiative decay rate can be derived in a different way from the estimation described in section 5. When we use an average $\Gamma_{\text{NR}}$ of 0.022 ns^-1^, the line fitting by equation (11) to the experimental data in Fig. 5(b) led to $\bar{\xi}\Gamma_{\text{R}}^{0}$ ~ 0.6 μs^-1^. If we use $\bar{\xi}$ = 0.1, $\Gamma_{\text{R}}^{0}$ ~ 6 μs^-1^ (its corresponding lifetime is 170 ns). This rate is in good agreement with the average $\Gamma_{\text{R}}^{0}$ of ~9 μs^-1^ shown in Table S1, which is estimated from the PL intensity and its decay rate without assuming the Purcell theory. Concerning the intrinsic radiative decay rate of the Cu-IECs, we found only a few reports, in which the rate is of the order of 0.01 μs^-1^ [S1, S12]. This means that the intrinsic radiative decay rate of the Cu-IECs in our system is three orders of magnitude larger than that in previous reports. Although further investigation is required, the radiative recombination could be accelerated by the superradiance effect, which takes place in an emitter ensemble localized in a sub-wavelength-sized volume with small spectral broadening [S13, S14].

**Table S1 Characteristic parameters of on- and off-resonant cavities, and an unpatterned Si film.** *s* is the position shift of the end holes of the L3 cavity. *Q*, *V*, and *β* are calculated by FDTD simulations. *K* is experimentally obtained by PL intensity measurements. $\Gamma_{\text{R}}^{0}$ and Γ_NR_ are estimated from the FDTD results, the PL intensities, and its decay rates obtained by PL measurements.

| *s* | *Q* | *V* (μm^3^) | *β* | *K*_on_ | *K*_off_ | $\Gamma_{\text{R}}^{0}$(ns^-1^) | Γ_NR_ (ns^-1^) |
| --- | --- | --- | --- | --- | --- | --- | --- |
| 0.08 *a*^*^ | 450 | 0.042 | 0.071 | 3.9 | 1.3 | 0.004 | 0.020 |
| -0.07 *a* | 3,300 | 0.029 | 0.054 | 7.1 | 0.73 | 0.002 | 0.020 |
| 0.03 *a* | 7,200 | 0.032 | 0.10 | 1.2 | 0.26 | 0.022 | 0.023 |
| 0.10 *a* | 16,000 | 0.035 | 0.044 | 2.2 | 0.62 | 0.006 | 0.023 |
| Off resonant cavity | - | 0.73 ^†^ | 0.077 | - | - | - | 0.022 ^‡^ |
| Unpatterned | - | 0.94 ^†^ | 0.0038 | - | - | 0.009 ^‡^ | 0.022 |

^*^ This results from the second-order mode of the L3 cavity. The others result from the fundamental mode.

^†^ The volume is equal to the Si volume excited by the laser with a spot diameter of ~2 μm.

^‡^ The decay rate is the average rate observed in the cavities.

**Figure S1 Quantum efficiency ratios and radiative decay rate ratios depending on normalized *Q*/*V*_c_. These ratios are normalized by the values for an unpatterned Si film.** (a) Enhancement and suppression ratios of the emission QE of the Cu-IECs in the on- and off-resonant cavities, respectively. The slightly sloped curve indicates the theoretical curve for the on-resonant cavity. The details for the curve are explained in the text. (b) Radiative decay rate ratios for the on- and off-resonant cavities. The sloped red line shows the fitted line for the on-resonant cavity. All of the horizontal solid blue lines show the average values for the off-resonant cavity. The horizontal broken lines indicate unity.

**References**

[S1] Watkins, S. P., Ziemelis, U. O., Thewalt, M. L. W. & Parsons, R. R. Long lifetime photoluminescence from a deep centre in copper-doped silicon. *Solid State Commun.* **43**, 687-690 (1982).

[S2] Fujita, M., Gelloz, B., Koshida, N. & Noda, S. Reduction in surface recombination and enhancement of light emission in silicon photonic crystals treated by high-pressure water-vapor annealing. *Appl*. *Phys*. *Lett*. **97**, 121111 (2010).

[S3] Fujita, M., Takahashi, S., Tanaka, Y., Asano, T. & Noda, S. Simultaneous inhibition and redistribution of spontaneous light emission in photonic crystals. *Science* **308**, 1296-1298 (2005).

[S4] Yosie, T. *et al*. Vacuum Rabi splitting with a single quantum dot in a photonic crystal nanocavity. *Nature* **432**, 200-203 (2004).

[S5] Wang, Q., Stobbe, S. & Lodahl, P. Mapping the local density of optical states of a photonic crystal with single quantum dots. *Phys. Rev. Lett.* **107**, 167404 (2011).

[S6] Gérard, J. M. *et al.* Enhanced spontaneous emission by quantum boxes in a monolithic optical microcavity. *Phys. Rev. Lett.* **81**, 1110-1113 (1998).

[S7] Steger, M. *et al*. Reduction of the linewidths of deep luminescence centers in ^28^Si reveals fingerprints of the isotope constituents. *Phys. Rev. Lett.* **100**, 177402 (2008).

[S8] Gayral, B., Gérard, J.-M., Sermage, B., Lemaître, A. & Dupuis, C. Time-resolved probing of the Purcell effect for InAs quantum boxes in GaAs microdisks. *Appl. Phys. Lett.* **78**, 2828-2830 (2001).

[S9] Fang, W. et al. Large enhancement of spontaneous emission rates of InAs quantum dots in GaAs microdisks. *Opt. Lett.* **27**, 948-950 (2002).

[S10] Pitanti, A., Ghulinyan, M., Navarro-Urrios, D., Pucker, G. & Pavesi, L. Probing the spontaneous emission dynamics in Si-nanocrystals-based microdisk resonators. *Phys. Rev. Lett.* **104**, 103901 (2010).

[S11] Purcell, E. M. Spontaneous emission probabilities at radio frequencies. *Phys. Rev.* **69**, 681 (1946).

[S12] Vinh, N. Q., Phillips, J., Davies, G. & Gregorkiewicz, T. Time-resolved free-electron laser spectroscopy of a copper isoelectronic center in silicon. *Phys. Rev. B* **71**, 085206 (2005).

[S13] Knoester, J. Optical dynamics in crystal slabs: Crossover from superradiant excitons to bulk polaritons. *Phys. Rev. Lett.* **68**, 654-657 (1992).

[S14] Greiner, C., Boggs, B. & Mossberg, T. W. Superradiant emission dynamics of an optically thin material sample in a shot-decay-time optical cavity. *Phys. Rev. Lett.* **85**, 3793-3976 (2000).
